# Extension of Nakagawa & Schielzeth's *R*^2^_GLMM_ to random slopes models

**DOI:** 10.1111/2041-210X.12225

**Published:** 2014-07-23

**Authors:** Paul CD Johnson

**Affiliations:** Institute of Biodiversity, Animal Health and Comparative Medicine, University of GlasgowGraham Kerr Building, Glasgow, G12 8QQ, UK

**Keywords:** coefficient of determination, generalized linear mixed model, random slopes model, random regression

## Abstract

1. Nakagawa & Schielzeth extended the widely used goodness-of-fit statistic *R*^2^ to apply to generalized linear mixed models (GLMMs). However, their *R*^2^_GLMM_ method is restricted to models with the simplest random effects structure, known as random intercepts models. It is not applicable to another common random effects structure, random slopes models.
2. I show that *R*^2^_GLMM_ can be extended to random slopes models using a simple formula that is straightforward to implement in statistical software. This extension substantially widens the potential application of *R*^2^_GLMM_.

## Introduction

The coefficient of determination, *R*^2^, is a widely used statistic for assessing the goodness-of-fit, on a scale from 0 to 1, of a linear regression model (LM). It is defined as the proportion of variance in the response variable that is explained by the explanatory variables or, equivalently, the proportional reduction in unexplained variance. Unexplained variance can be viewed as variance in model prediction error, so *R*^2^ can also be defined in terms of reduction in prediction error variance. Insofar as it is justifiable to make the leap from ‘prediction’ to ‘understanding’, *R*^2^ can be intuitively interpreted as a measure of how much better we understand a system once we have measured and modelled some of its components.

*R*^2^ has been extended to apply to generalized linear models (GLMs) (Maddala 1983) and linear mixed effects models (LMMs) (Snijders & Bosker 1994) [reviewed by (Nakagawa & Schielzeth 2013)]. Nakagawa & Schielzeth (2013) proposed a further generalization of *R*^2^ to generalized linear mixed effects models (GLMMs), a useful advance given the ubiquity of GLMMs for data analysis in ecology and evolution (Bolker *et al.* 2009). A function to estimate this *R*^2^_GLMM_ statistic, *r.squaredGLMM*, has been included in the *MuMIn* package (Bartoń 2014) for the *R* statistical software (R Core Team 2014). However, Nakagawa and Schielzeth's *R*^2^_GLMM_ formula is applicable to only a subset of GLMMs known as random intercepts models. Random intercepts models are used to model clustered observations, for example, where multiple observations are taken on each of a sample of individuals. Correlations between clustered observations within individuals are accounted for by allowing each subject to have a different intercept representing the deviation of that subject from the global intercept. Random intercepts are typically modelled as being sampled from a normal distribution with mean zero and a variance parameter that is estimated from the data. Although random intercepts are probably the most popular random effects models in ecology and evolution, other random effect specifications are also common, in particular random slopes models, where not only the intercept but also the slope of the regression line is allowed to vary between individuals. Random intercepts and slopes are typically modelled as normally distributed deviations from the global intercept and slope, respectively. For example, random slopes models, under the name of ‘random regression’ models, are used to investigate individual variation in response to different environments (Nussey, Wilson & Brommer 2007). The aim of this article is to show how Nakagawa and Schielzeth's *R*^2^_GLMM_ can be further extended to encompass random slopes models.

## Nakagawa and Schielzeth's *R*^2^_GLMM_

Nakagawa & Schielzeth (2013) defined two *R*^2^ statistics for GLMMs, marginal and conditional *R*^2^_GLMM_, that allow separation of the contributions of fixed and random effects to explaining variation in the responses. Marginal *R*^2^_GLMM_ gauges the variance explained by the fixed effects as a proportion of the sum of all the variance components: eqn 1
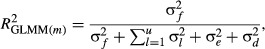
where 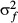 is the variance attributable to the fixed effects, 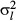 is the variance of the *l*th of *u* random effects, 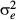 is the variance due to additive dispersion and 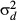 is the distribution-specific variance. The residual variance, 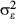, is defined as 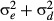 for the purposes of this manuscript but see Nakagawa & Schielzeth (2013) for an alternative definition of dispersion. Conditional *R*^2^ additionally includes in the numerator the variance explained by the random effects: eqn 2
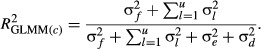


It is the definition of the random effect variances, the 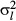, that requires generalization to allow *R*^2^_GLMM (m)_ and *R*^2^_GLMM (c)_ to be extended beyond random intercepts models. In Nakagawa and Schielzeth's formula, 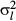 is simply the variance of the *l* th random intercept. This formula is correct for random intercept models because each observation has the same random effect variance. However, in other random effects specifications, the random effect variance can differ between observations, and, as pointed out by Nakagawa and Schielzeth, this causes difficulties in computing a single random effect variance component.

## Extension of *R*^2^_GLMM_ to random slopes models

Consider the simplest and most familiar random slopes GLMM, a LMM with a single random intercept and a single random slope: eqn 3
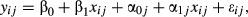

eqn 4
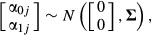

eqn 5
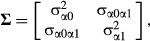

eqn 6
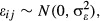
where *Y*_*ij*_ and *x*_*ij*_ are, respectively, the response and predictor values (covariates) for the *i*th observation on the *j*th individual. Random deviation of the *j*th individual from the fixed global intercept, *β*_0_, is represented by α_0*j*_, while random deviation from the fixed global slope, *β*_1_, is represented by α_1*j*_. Because intercepts and slopes are typically correlated, three parameters are required to model the random effect, which are represented by the covariance matrix **Σ.** The leading diagonal of **Σ** consists of the random intercept variance, 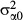, and the random slope variance, 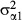, while the off-diagonal element is the covariance, σ_α0α1_, between the random intercept and random slope. Finally, *ɛ*_*ij*_ is the residual of the *i*th observation on the *j*th individual and 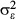 is the residual variance. For LMMs, 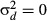, so that 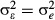.

The difficulty of defining 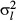 for this model arises from the dependence of the random effect variance component on *x*_*ij*_, which implies that 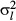 cannot be defined from **Σ** alone, but requires input from the *x*_*ij*_. An observation-specific random effect variance, 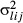, can be defined, given *x*_*ij*_, as eqn 7
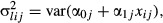
showing the dependence of 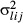 on *x*_*ij*_. For example, when *x*_*ij*_ = 0 (i.e. at the intercept),
eqn 8
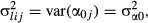
while when *x*_*ij*_ = 1, eqn 9
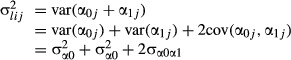
(Snijders & Bosker 2012). In the most extreme case where the *x*_*ij*_ values are unique, there will be as many random effect variances as observations. The first step to estimating the random effect variance component is to estimate each 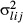. The random effect portion of the model, α_0*j*_ + α_1*j*_*x*_*ij*_, can then be viewed as a mixture of *n* normal distributions with a common mean of zero but up to *n* different variances, where *n* is the number of observations. When the mean is constant, the variance of a mixture is simply the mean of the individual variances (Behboodian 1970). The mean random effect variance is therefore eqn 9
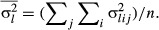
x

A simple and general formula for 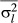 given any value of *x*_*ij*_ can be derived as follows. For any random effects specification, let **Z** be the design matrix of the random effects of a GLMM with *n* rows and *k* columns corresponding to the *k* random effects, and **Σ** the covariance matrix of the random effects of dimension *k*. For example, in the simple random slopes model in equations 3-6, the first column of **Z** is a vector of ones corresponding to the random intercept, while the second is the predictor variable, the *x*_*ij*_. The vector of observation-level random effect variances is the leading diagonal of the *n *× *n* matrix **ZΣZ**′, where **Z**′ is the transpose of **Z** (Laird & Ware 1982). The mean random effect variance, 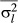, is the mean of this vector, that is, eqn 11
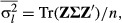
where the Tr denotes the trace operation, which sums the leading diagonal. An index notation version of the matrix notation equation 11 is contained within equation 20 of Snijders & Bosker (1994). The advantage of the matrix version is computational simplicity. Equation 11 gives the same results as Nakagawa & Schielzeth's method for random intercepts models but can also be used for random slopes models as well as models with no intercept. An estimate of 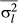 for use in Equations 1 and 2 can be easily computed from the estimated covariance matrix of the *l*th random effect. Examples of the application of this procedure to estimating *R*^2^_GLMM_ from random slopes GLMMs using R are provided as Data S1.

The Supplementary R code also illustrates a simplified method of estimating the term *β*_0_ in equation A6 of Nakagawa & Schielzeth (2013), which approximates 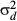 for a Poisson GLMM. Rather than refit the model after centring or dropping the covariates as recommended, *β*_0_ can be more easily estimated by taking the mean of 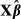, the linear predictor, where **X** is the design matrix for the fixed effects and 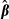 is the vector of fixed effect estimates.

These extensions to *R*^2^_GLMM_ have been incorporated into the *r.squaredGLMM* function in version 1.10.0 of the *MuMIn* package (Bartoń 2014).

## Discussion

The extension described above allows both marginal and conditional *R*^2^_GLMM_ to be estimated from a random slopes model, obviating the need to approximate *R*^2^_GLMM_ from the corresponding random intercepts model as recommended by Nakagawa & Schielzeth (2013). It is clearly preferable to estimate *R*^2^_GLMM_ from the correct model given that there is no computational cost but is the improvement in either marginal or conditional *R*^2^_GLMM_ likely to be substantial? Nakagawa & Schielzeth (2013) suggest that marginal and condition *R*^2^_GLMM_ will usually be very similar when approximated from a random intercepts fit, and Snijders & Bosker (2012) make a similar claim for their related *R*^2^_1_ and *R*^2^_2_ statistics. Not surprisingly, the gain in accuracy in both *R*^2^_GLMM_ statistics will depend on how well the random intercepts model approximates the random slopes model. The accuracy of the marginal *R*^2^_GLMM_ approximation will depend on the accuracy of the global slope (or slopes) estimate from the random intercepts model, because the scale of the global slope (or slopes) estimate determines 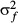 (Nakagawa & Schielzeth 2013), which in turn determines marginal *R*^2^_GLMM_. For balanced data, where the numbers of observations and the covariate distributions are balanced between groups, this approximation should be good, so the estimates of the global slope and marginal *R*^2^_GLMM_ are likely to be very similar under both models. However, unbalanced data are common in ecology, for example where sampling strategies are constrained in space by variable access to sampling sites or in time by fluctuating resources, and in such cases the improvement in marginal *R*^2^_GLMM_ could be considerable. For example, if one individual (or site, etc.) yields an unusually large number of observations, the global slope estimate will be biased towards that individual in a random intercepts model but not in a random slopes model. Examples of both scenarios are given in the Supplementary R code (Data S1).

Improvement in conditional *R*^2^_GLMM_ is easier to predict and explain. Regardless of the adequacy of the marginal *R*^2^_GLMM_ approximation, if the random slopes model fits substantially better than the random intercepts model, it should have lower residual variance (or less overdispersion, in the context of overdispersed Poisson or binomial GLMMs) and therefore higher conditional *R*^2^_GLMM_.

This extension will apply to other statistics that incorporate a random effects variance component calculated from a random slopes model, including the intraclass correlation coefficient (ICC), which gauges variance between groups (e.g. individuals or sites) as a proportion of the total variance. ICC can be used to measure intraindividual repeatability, also known as consistency, and has been applied widely in ecology and evolutionary biology (Nakagawa & Schielzeth 2010). Like *R*^2^, ICC has also been generalized to random intercepts GLMMs by Nakagawa & Schielzeth (2010), but not to random slopes GLMMs. Equation 11 could also be applied to calculating repeatability (Nakagawa & Schielzeth 2010) by fixing a column of **Z** to a single value. For example, age dependence in phenotypic consistency could be investigated by estimating ICC conditioned on a range of ages.

In conclusion, the extension of *R*^2^_GLMM_ to random slopes GLMMs substantially widens the range of models to which this useful measure can be applied.

## Data accessibility

R scripts: uploaded as online supporting information.

## Supporting Information

Data S1 R code illustrating the calculation of R^2^_GLMM_.
